# Assessing the disparity in spatial access to hospital care in ethnic minority region in Sichuan Province, China

**DOI:** 10.1186/s12913-016-1643-8

**Published:** 2016-08-17

**Authors:** Xiuli Wang, Jay Pan

**Affiliations:** 1 Department of Environment, College of Architecture and Environment, No.24 South Section 1, Yihuan Road, Chengdu, 610065 China; 2 West China School of Public Health, Sichuan University, No.17 People’s South Road, Chengdu, 610041 China

**Keywords:** Spatial accessibility, Hospital care, Enhanced two-step floating catchment area method, Ethnic minority region, Planning, China

## Abstract

**Background:**

There is a great disparity in spatial accessibility to hospital care between ethnic minority and non-minority regions in China. Being one of the basic social demands, spatial access to hospital care in minority regions draws increasing attention in China in recent years. We performed this study to have a better understanding of spatial access to hospital care in ethnic minority region in China, and to guide the allocation of government investment in the future.

**Methods:**

Sichuan Province, southwest of China was selected as a sample to examine the difference in hospital access between ethnic minority and non-minority region in China. We applied the shortest path analysis and the enhanced two-step floating catchment area (E2SFCA) method under ArcGIS 9.3 environment.

**Results:**

In Sichuan, healthcare access in ethnic minority region is worse than in non-minority region in terms of time to hospital and the value of spatial accessibility. There is relatively greater inequality in access to doctors and health professionals than in access to hospital beds. In ethnic minority region, the balance between primary, secondary, and tertiary hospitals, as well as between public and private hospitals, is less even, compared with the non-minority region. The disparity within ethnic minority region is larger than in non-minority region.

**Conclusions:**

The combination of shortest path analysis and E2SFCA method is superior to the traditional county ratio method in assessing spatial access to healthcare. Compared to the non-minority region, ethnic minority region rely more heavily on government investment to provide healthcare. In ethnic minority region, the current distribution of primary, secondary and tertiary hospitals is inappropriate, and there is an urgent shortage of healthcare personnel. We therefore recommend that the government use preferential policies to encourage more social capital investment in ethnic minority region, use government investment as a supplement to build a more equitable healthcare market, encourage doctors to work in such regions, and push forward road construction in rural area.

## Background

Since the establishment of the People’s Republic of China (PRC) in 1949, the Chinese Communist Party (CCP) has aimed to build a socialistic society with justice and equality [1, 2]. Among the 56 ethnic groups in China, the Han is the ethnic majority, accounting for more than 90 % of China’s population. They live in almost every region in China and own most property, technology, and resources [1–5]. The ethnic minority communities, due to historical reasons, are more concentrated in remote, under-developed areas [2, 6]. To promote equality between ethnic groups, the Chinese government has implemented many policies to support ethnic minorities. Being classified as one of the 55 ethnic minorities bestows advantages in family planning (many minority groups are exempted from the one-child policy), in education (minorities have preferential treatment regarding entry to higher education, and compulsory education in Tibet is 12 years, longer than the rest of China), in employment, in business development, and in political representation [7–10]. The Chinese government has set up minority regions (including minority autonomous regions, minority counties, and minority villages) based on proportion of minority population, historical factors, local customs and culture, to offer efficient financial and political supports. Minority autonomous regions are the most concentrated habitation of ethnic minority population, with well-preserved custom and culture. Also in minority autonomous regions, ethnic minority constitute the majority of local government, and has many self-governance rights.

Effective allocation of healthcare resources can reduce health inequalities among Chinese citizens, thus ensuring economic development and social stability. The Chinese government recognized this in its 2009 healthcare reform, when it established “the progressive equalization of the basic public health services” as one of its five key projects. In China’s current health care delivery system, hospitals play a leading role. According to China’s Statistical Yearbook of Health, nearly 60 % of outpatient and emergency treatment, and more than 70 % of inpatient treatment was provided by hospitals in 2012. Hence, differences in spatial access to hospitals represent a considerable barrier to the government’s equity goals: in 2010, 34.6 % of rural respondents to a national survey (83.87 million people) cited distance as a major barrier in getting timely hospital treatment versus only 1.37 % of urban residents [11]. Ethnic minorities are particularly vulnerable to such inequalities, since many live in remote, rural regions.

In China, population-to-provider ratio (PPR) is the preferred method for characterizing the accessibility of healthcare services because it is easy to implement and understand [12]. However, it does not reflect the real world as accessibility differs inside the administrative unit, and cross-border service-seeking behaviors exist [13]. A number of new techniques have been developed based on the gravity model, of which the two-step floating catchment area (2SFCA) method has gained the most traction recently [14]. In the 2SFCA method, catchment areas for every supply and demand point are calculated and the capacity is distributed in the supply point’s catchment area; when this falls into the demand point’s catchment area, the capacity can be determined by the certain demand point [12]. The development of Geographical Information System (GIS) makes the 2SFCA method easy to implement, and several modifications and extensions have been suggested, like the enhanced two-step floating catchment area (E2SFCA) method [15] which applies multiple catchments.

In this study, we applied the E2SFCA method to evaluate the spatial accessibility of hospital-based services in Sichuan Province, a region with a large, multi-ethnic population. Our intention was to compare the accessibility of hospital care between ethnic minority region and non-minority region in Sichuan, analyze why any disparities exist, and provide scientific suggestions to policy makers and planners for adjusting and reallocating hospital resources.

## Methods

### Study area

**Fig. 1:**
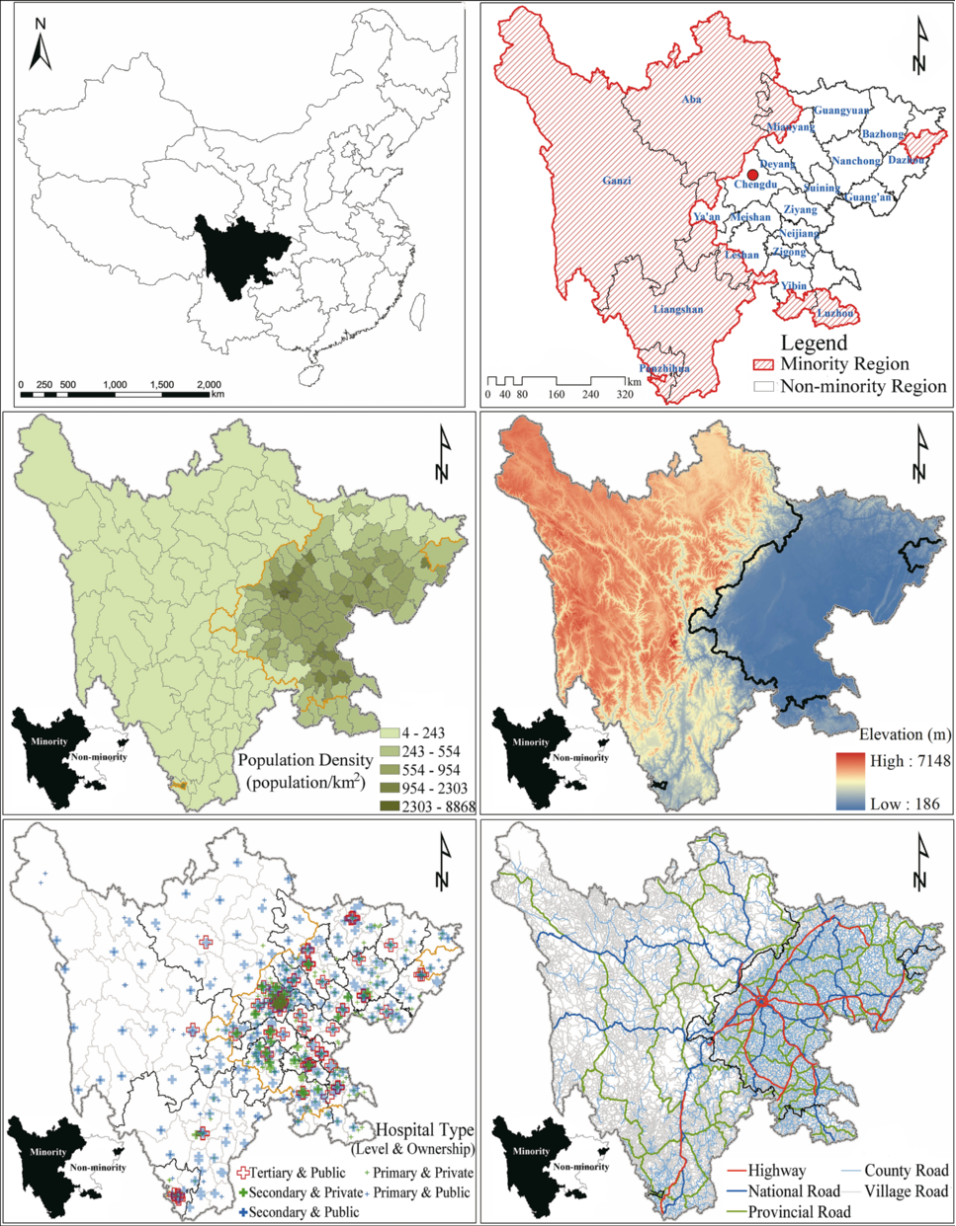
Population, transportation, ethnic minority region, and hospital distribution in Sichuan Province, 2012. Note: Based on the data collected from the Health and Family Planning Commission of Sichuan Province (hospital data), the National Geomatics Center of China (digital elevation model, administrative boundary and road network data), and the department of the Statistics Bureau of Sichuan (population and county type data), the listed maps were developed under ArcGIS 9.3 environment

**Fig. 2:**
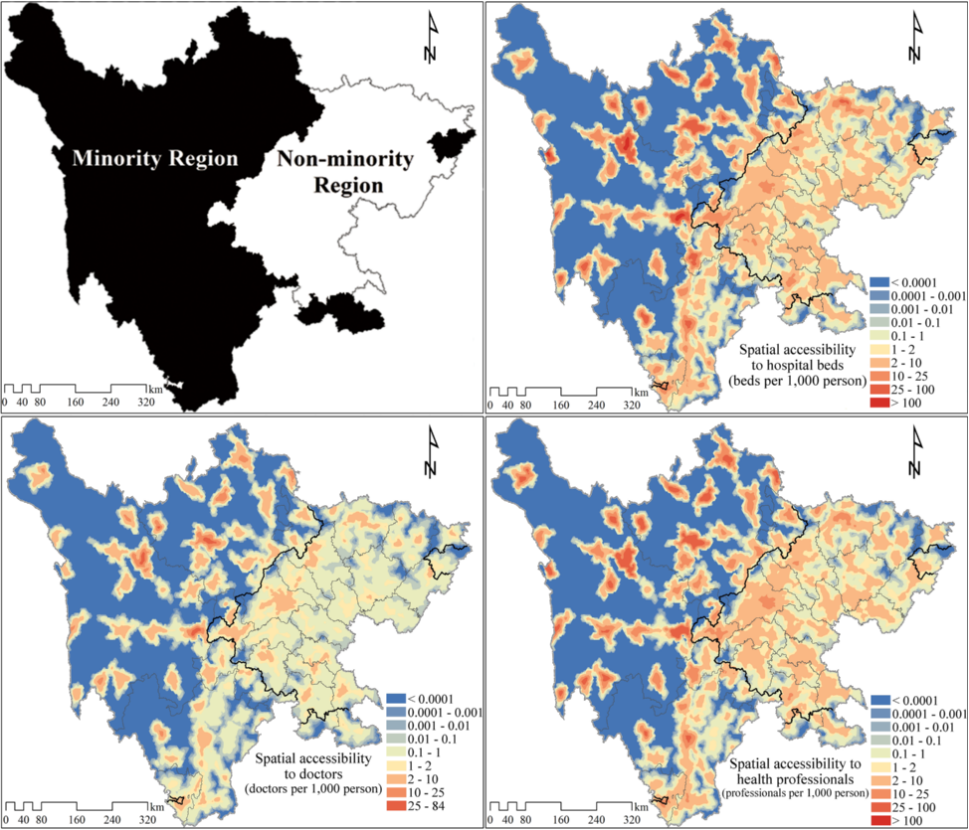
Spatial accessibility of hospital beds, doctors and health professionals by E2SFCA method. Note: E2SFCA, enhanced two-step floating catchment area. Based on the spatial access to all hospitals, all doctors, and all health professionals of each population point, a simple Kriging interpolation method was used to produce these contour maps. Different values for the impendence coefficient of Gaussian function were also trailed (impendence coefficient value of 740 providing weights values of 0.967, 0.582, 0.065 for the 0–10, 10–30, and 30–60 min subzones, respectively, and 1040 providing weights values of 0.976, 0.681, 0.143 for the 0–10, 10–30, and 30–60 min subzones, respectively). However, only minimal difference to overall accessibilities were found, and the disparity between the ethnic minority and non-minority regions were consistent among the different weights settings

Sichuan Province (92°21′ ~ 108°12′E and 26°03′ ~ 34°19′N) was selected as a sample to examine the difference in hospital access between ethnic minority and non-minority region in China. Sichuan Province is the 5^th^ largest province in China with a total area of 486,000 km^2^ and a population of 90.9 million (in 2012). Sichuan can be roughly divided into two geographic/demographic zones: west Sichuan, which is a vast, rural, mountainous, sparsely populated terrain of high altitude; and east Sichuan, which is a densely populated plain, with a well-developed road network and economy (Fig. 1). This geographic dichotomy also broadly mirrors the situation in the nation as a whole. In Sichuan, 13.7 million residents live in the 340,900 km^2^ ethnic minority region (“west Sichuan”), while the non-minority region is smaller at 146,200 km^2^ and holds 77.2 million people (the black line in Figs. 1 and 2 indicates the boundary between ethnic minority and non-minority region in Sichuan Province). Even in ethnic minority region, minority population are more concentrated in rural areas, while the Han ethnic majority living in minority region are more concentrated in urban areas (Table 1).

**Table 1:** Minority population in the three autonomous regions in Sichuan Province (Million)

National Autonomous Region	Urban Areas			Rural Areas		
	Minority population	Total population	Proportion of minority	Minority population	Total population	Proportion of minority
Aba	0.16	0.27	59.26 %	0.68	0.90	75.56 %
Ganzi	0.11	0.21	52.38 %	0.89	1.09	81.65 %
Liangshan	0.29	1.25	23.20 %	2.38	4.54	52.42 %

Note: Data were extracted from the tabulation on the 2010 population census of Sichuan Province

### Data

We acquired the data for hospitals, road networks, population, county type and administrative boundaries for analysis, with sources and detailed information listed in Table 2. All applied data were extracted by corresponding government departments or from publicly published yearbooks, and no human participants were involved in this study.

**Table 2:** Sources and detailed information of basic data

Data name	Data type	Source	Information
Hospital	Point	The Health and Family Planning Commission of Sichuan Province	Name Address (longitude and latitude) Type (public or private) Level (primary, secondary, and tertiary) Capacity (number of hospital beds, doctors, and health professionals)
Road network	Line	The National Geomatics Center of China	Shape Type (highway, state road, provincial road, county road, village road)
Population	Table	The Department of the Statistics Bureau of Sichuan	Number (population of every county-level administration unit)
Administrative boundary	Line	The National Geomatics Center of China	Name Shape
County type	Table	The Department of the Statistics Bureau of Sichuan	Type (ethnic minority or non-minority)

Three indicators were used to characterize the capacity of hospitals: the number of hospital beds, the number of health professionals, and the number of doctors. Doctors (include both registered doctors and assistant doctors), as clarified in the National Health and Family Planning statistical yearbook, is part of health professionals, which consists of registered doctors, assistant doctors, registered nurses, pharmacists, and laboratory technicians.

Hospitals of different ownership and levels were considered in this study. Public hospitals are supported by the government, and private hospitals are managed by social investors. The three levels of hospitals are divided according to the National Health and Family Planning Commission of the People’s Republic of China. From primary, secondary to tertiary hospitals, there is an increase in hospitals beds, facility, function, and service quality. The primary hospitals are community health centers or hospitals with less than 100 beds, which offer basic health care services for residents living in the community. Thus primary hospitals are most widely distributed, located in rural villages, urban communities and streets. The secondary hospitals are regional hospitals covering several communities. Apart from offering medical and health services, they also have some teaching and researching missions. The secondary hospitals are usually located in towns. The tertiary hospitals are the best equipped, they offer specialized health services for population from different regions, execute higher education and research tasks, and usually have more than 500 hospital beds. The tertiary hospitals are mostly located in counties and cities.

### Study variables

The shortest travel time to a hospital and the spatial accessibility of healthcare services (including hospital beds, doctors, and health professionals) were the outcome variables. We calculated spatial accessibility for hospitals of different ownership (public vs. private) and different administrative level (primary, secondary, tertiary), separately and then combined. The shortest travel time was calculated using the nearest-neighbor method, which considers the time boundary between a given location (patient) and the nearest hospital. The spatial accessibility was more complicated; it considered all hospitals within the reachable range of a location, since patients can have options but also need to compete with other patients for the limited capacity of hospitals. This was achieved through the E2SFCA method.

### The nearest-neighbor method

Both the nearest-neighbor method and the E2SFCA method were realized under an ArcGIS 9.3 (ESRI, 2009) platform. We assumed that population density in each county was even and calculated a grid map (grid size is 2 km*2 km). This method was chosen because a higher resolution of population was not available for the study area. The road network was loaded into ArcGIS and each road was given a standard speed, 120 km/h for highway, 100 km/h for state road, 80 km/h for provincial road, 60 km/h for county road and 40 km/h for village road, which were derived from the Highway Technical Standards of China, based on road class, traffic, and actual physical conditions. The closest facility command under network analysis tools in ArcGIS was then used to calculate the closest hospitals to each population grid and the time boundary. The shortest travel time to hospitals were classified into 5 categories, 0–10, 10–30, 30–60, 60–120, and > 120 min. The threshold of 120 min was used in this study, because a significant proportion of area and population were not located within a 60 min range of the hospitals. Which is usually referred to as the Golden Hour (the time period following a traumatic injury during which there is the highest probability that medical treatment will prevent death). Adding a 60–120 min subzone helped to demonstrate the variability in accessibility within the study region. Moreover, the “subgroup” analysis identifies the under-served (60–120min) regions and the extremely under-served (>120 min) regions, respectively, which provides evidence for the related policy making.

### The E2SFCA method

The E2SFCA method includes two major steps. The first step is to calculate the supply-to-demand ratio *R*_*j*_ of every hospital *j* based on equation 1. The medical capacity of each hospital (*S*_*j*_) is shared by the population in several sub-zones around it. *d*_*kj*_ represents the travel cost between *j* and *k*, *D*_*r*_ is the *r*^th^ sub-zone based on travel time, *W*_*r*_ is a distance-weight for *D*_*r*_, which is based on a distance decay function and usually calculated using the Gaussian function, inverse-power function, or exponential function. The second step is to calculate the spatial accessibility *A*_*i*_^*F*^ of each population unit *i* through equation 2. *R*_*k*_ is the supply-to-demand ratio of hospital *k*, and hospital site *k* falls in different sub-zones of population unit *i* is given different *W*_*r*_ values as in the first step.

1 $$ {R}_j=\frac{S_j}{{\displaystyle {\sum}_{j\in \left({d}_{kj}\in {D}_r\right)}{P}_k{W}_r}}=\frac{S_j}{{\displaystyle {\sum}_{j\in \left({d}_{kj}\in {D}_1\right)}{P}_k{W}_1+{\displaystyle {\sum}_{j\in \left({d}_{kj}\in {D}_2\right)}{P}_k{W}_2}+{\displaystyle {\sum}_{j\in \left({d}_{kj}\in {D}_3\right)}{P}_k{W}_3}}} $$

2 $$ {A}_i^F={\displaystyle {\sum}_{i\in \left({d}_{ik}\in {D}_r\right)}{R}_k{W}_r}={\displaystyle {\sum}_{i\in \left({d}_{ik}\in {D}_1\right)}{R}_k{W}_1}+{\displaystyle {\sum}_{i\in \left({d}_{ik}\in {D}_2\right)}{R}_k{W}_2}+{\displaystyle {\sum}_{i\in \left({d}_{ik}\in {D}_3\right)}{R}_k{W}_3} $$

In our study, we used hospital beds, doctors, and other health professionals as medical capacity, generated three sub-zones (0–10, 10–30, and 30–60 min) for each hospital and population unit (the same 2 km*2 km population grid map as in the nearest-neighbor method), and calculated distance-weight based on the Gaussian function (the impendence coefficient value was set to 440, and provided the weight values 0.945, 0.403, 0.010 for the 1^st^, 2^nd^, and 3^rd^ subzone respectively) [15, 16]. The 10, 30, and 60 min were used as cut-off values. Because 10 min is usually viewed as initial impendence that presents as small barrier to accessing hospital care, while 30 min is viewed as standard travel time advocated by various health plans in China, and a larger catchment size (60 min) is helpful to incorporate isolated areas in rural area [17, 18]. The weight for the 30–60 min subzone (0.010) is relatively small, because there is an increasing barrier in access to healthcare services as the travel time increases [17] and the 30–60 min subzone is the outermost subzone before the contribution of facilities got zero. Because of the complexity of Sichuan Province (population density, urban/ rural area, ethnic minority/ non-minority region, geographic characteristics, et al.), and for comparison between ethnic minority and non-minority regions, the same sub-zones and weightings were used throughout the research area. We assumed healthcare service seeking behavior in different regions in Sichuan to be the same because in a well-developed healthcare market the time costs of being served should be equal.

### Statistical analyses

SPSS 20.0 and Excel 2010 were used for statistical analyses.

## Results

### The shortest travel time to hospitals

Table 3 presents the percentage of area and population which fall within the concentric time zones around each hospital. The distribution of hospitals in the non-minority region is more reasonable and convenient to residents. The percentage of residents living within 10 min of a hospital in the non-minority region (6.92 %) is four times that of ethnic minority region (1.71 %), while the percentage of residents living more than 120 min (indicating extremely under-served) away from the nearest hospital is about half (5.73 % versus 11.55 %). In the non-minority region, more than 70 % of people live 10–60 min away from the nearest hospital, but in ethnic minority region, most people live 30–120 min from hospitals. In ethnic minority region, except for journey times in excess of 120 min, the percentage area within a certain journey time is less than the percentage of the population living there. In the non-minority region, the percentage of population exceeds the percentage of area when travel time is less than 30 min, while in the rest sub-zones (30–60, 60–120, and >120 mins), the percentage of population turns out to be less than the percentage of the area.

**Table 3:** Percentage of area and population in different time zones from the closest hospital

Shortest Travel Time (minutes)		Ethnic minority region			Non-minority region		
		All Hospitals	Public Hospitals	Private Hospitals	All Hospitals	Public Hospitals	Private Hospitals
0–10	Area (%)	1.00	0.86	0.46	5.21	3.95	3.83
	Population (%)	1.71	1.37	0.94	6.92	5.36	5.39
10–30	Area (%)	7.49	7.09	2.73	32.18	28.37	27.07
	Population (%)	14.19	12.57	7.53	36.62	32.92	32.21
30–60	Area (%)	19.40	19.31	6.69	39.50	41.41	38.86
	Population (%)	35.16	34.68	21.56	37.14	40.03	37.44
60–120	Area (%)	36.74	37.30	12.61	17.94	20.96	19.54
	Population (%)	37.39	39.67	33.63	13.59	15.87	12.60
>120	Area (%)	35.38	35.44	77.50	5.16	5.31	10.70
	Population (%)	11.55	11.72	36.34	5.73	5.82	12.37

Across the whole of Sichuan, public hospitals serve a greater population and area than private hospitals within a 2-h distance. In the non-minority region, the area and population served by public hospitals and private hospitals are comparable, however the disparity grows as travel time increases from 0 to 120 min. In the minority region, the service area covered by public hospitals is generally more than twice that of the private hospitals, and the population is 1.2 to 1.7 times greater.

### Spatial accessibility of healthcare services

Accessibility rates are listed in Table 4 and Fig. 2. They demonstrate that healthcare services are more accessible in the non-minority region. Accessibility for doctors, health professionals, and beds are all higher in the non-minority region than in ethnic minority region, although the inequality in hospital beds (1.49 times) is smaller than the inequality in doctors and health professionals (1.58 times). Hospitals of different ownership (public and private) and level (primary, secondary, and tertiary) show the same trend except for primary and secondary public hospitals. The three accessibility indicators are all slightly higher for secondary public hospitals in ethnic minority region than they are in non-minority region. In primary public hospitals, accessibility to doctors is marginally higher in ethnic minority region while accessibility rates for health professionals and beds are both lower.

**Table 4:** Spatial accessibility of hospital services in minority and non-minority region

Hospital Capacity	Region	All Hospitals	Public Hospitals			Private Hospitals	
			Primary	Secondary	Tertiary	Primary	Secondary
Doctors	Minority	0.57	0.07	0.37	0.09	0.04	0.00
	Non-minority	0.90	0.06	0.34	0.33	0.14	0.03
Health Professionals	Minority	1.81	0.18	1.19	0.30	0.13	0.01
	Non-minority	2.86	0.19	1.05	1.07	0.45	0.09
Hospital Beds	Minority	2.26	0.25	1.49	0.29	0.22	0.02
	Non-minority	3.37	0.34	1.22	1.07	0.64	0.10

Note: To get the spatial accessibility (including spatial access to doctors, health professionals, and hospitals beds) of different types of hospitals, hospitals of different ownership and level were firstly calculated using the E2SFCA (enhanced two-step floating catchment area) method separately. The spatial accessibility of population points located in ethnic minority and non-minority region were then weight averaged with population density respectively to get the average spatial accessibility of ethnic minority and non-minority region

Healthcare service distribution differs between the minority and non-minority region by hospital ownership type and level. In the non-minority region, private hospitals account for a much greater share of all services. For instance, the accessibility of all doctors in the non-minority region is 0.90 (see Table 4), of which 0.17 are provided by private hospitals (0.14 provided by doctors of private primary hospitals, 0.03 provided by doctors of private secondary hospitals, and because there is no private tertiary hospital, the accessibility of private tertiary doctors is 0. The total accessibility of private hospital doctors is the sum of private primary doctors and private secondary doctors. In this case, the number is 0.17). Of all doctor accessibility in the non-minority region, about 19 % (0.17/0.90) were provided by private hospital doctors, which is 2.7 times higher than in ethnic minority region (versus 2.4 times for health professionals and 2.2 times for beds). And at the primary level, most accessibility were actually provided by private hospitals in the non-minority region (e.g. the accessibility of health professionals provided by private primary hospitals is 0.45 VS. 0.19 by public hospitals). Also healthcare services are more evenly distributed between hospitals of different levels in the non-minority region. Primary, secondary and tertiary hospitals provided approximately 25 %, 40 %, 35 % of total services (e.g. doctor accessibility of public primary hospitals (0.06) plus doctor accessibility of private primary hospitals (0.14) is the total doctor accessibility provided by primary hospitals, divided by the total doctor accessibility 0.90, comes the percentage of doctor accessibility provided by primary hospitals – 22 %, for health professional accessibility and hospital bed accessibility, the results are 22 % and 29 % respectively, and finally 25 % was chosen as the percentage of services provided by primary hospitals), while in ethnic minority region the values were 18 %, 65 % , and 17 %, respectively.

**Fig. 3:**
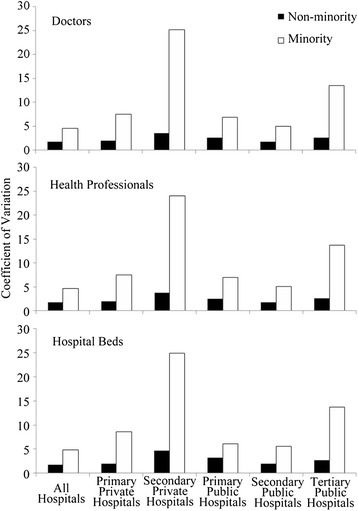
Residents’ coefficient of variation to doctors, health professionals and hospital beds in minority and non-minority region

As shown in Fig. 2, within the non-minority region there is less inequality in accessibility. To investigate intra-regional differences in accessibility, we used SPSS 20.0 to calculate the coefficient of variation (CV) of resident points in the two regions separately, and the results are presented in Fig. 3. In the non-minority region, CV values are lower than 5, while in ethnic minority region, CV values are much higher with a peak around 25 for secondary private hospitals.

## Discussion

In our study, we found that, across a wide range of indicators, the ethnic non-minority region in Sichuan has better access to healthcare services. The E2SFCA results are higher, indicating that residents in the non-minority region have more potential providers. The coefficient variations are lower, indicating that healthcare services are more evenly distributed. The percentage of residents and the total area covered within a two-hour service area of a hospital are higher, indicating greater convenience of service, although, in absolute terms, there is a larger number of people beyond the two-hour service area in the non-minority region due to much higher population density (13 times that of ethnic minority region).

In ethnic minority region, the shortage of personnel (doctors and health professionals) is more acute than the shortage of facilities (beds). The ethnic minority region in China are typically concentrated in remote mountainous areas, where transportation, economy, and technology are more limited (except regions with scenic spots, precious mineral mines/ medicinal plants) [19], which makes such regions less attractive to live in for doctors. Facilities, however, mostly depend on investment, and the government has devoted much more in ethnic minority region than in non-minority region to narrow the gap caused by limited social investment in ethnic minority region.

Apart from a shortage of hospitals in ethnic minority region, imbalances in the healthcare market there also need to be addressed. In ethnic minority region in Sichuan, about two thirds of hospitals are secondary, compared with around two fifths in non-minority region. The limited number of primary hospitals endangers access to the most basic healthcare services, while the shortage of tertiary hospitals restricts effective control of complex conditions. The tertiary hospital deficiency is concerning, since the literature demonstrates their key role in the Chinese health system: they have the best medical facilities and equipment, their doctors are relatively more qualified and experienced, they are more successful in controlling diseases [20, 21], and they receive the highest levels of investment [22].

Only a few private hospitals have been established in ethnic minority region, in contrast to the non-minority region where the private sector is the dominant provider of primary hospital services. Although the 2009 healthcare reform did much to facilitate private investment in healthcare, ethnic minority region still struggles to attract investors, owing to its sparse population, difficult geography and weak economy. Hence, to ensure equal hospital spatial accessibility, more public health funds need to be invested in ethnic minority region.

Intra-regional disparities in healthcare accessibility are much worse in ethnic minority region (Figs. 2 and 3). According to the spatial distribution of hospitals in Sichuan Province in 2012 (Fig. 1), the limited number of hospitals in ethnic minority region tend to be clustered around business and political centers, where transportation are also more convenient. The spatial accessibility in such centers tend out to be much higher than the rest area (even higher than most non-minority region while the rest area in ethnic minority area have practically no access at all). As is shown in Fig. 2, several locations with high spatial accessibility (shown in dark red) distributed within a large area with no hospital access (shown in dark blue) in the ethnic minority region. The coefficient of variation (Fig. 3) also indicated a larger disparity within ethnic minority regions than in non-minority region. In ethnic minority regions, Han people tend to live in cities, while ethnic minorities live in the countryside (Table 1). Hence, current patterns of government investment (concentrating in urban centers) may result in only limited gains to ethnic minority health, but an overall increase in health inequalities.

Our study assumed an even population density due to data limitation (which is not consistent with the real condition), the population density in ethnic minority regions is much lower compared with the non-minority regions, because one of the characteristics of ethnic minority region is few people living in vast area. Lower population density indicates less competition, and healthcare resources are clustered in business and political centers in ethnic minority region, resulting in the extreme high spatial accessibility in such areas. However, in cities or nearby main roads, the population density tend to be higher than average, while in area with steep slope and poor access, the population density are much lower (consistent with the distribution of hospitals) Thus the disparity of spatial accessibility within ethnic minority regions may have been overestimated. The actual accessibility is likely to be lower than our calculated results where population density is higher (e.g. cities in ethnic minority region), and higher than our estimation where population density is lower.

The same catchment sizes (0–10, 10–30, 30–60 min) and weightings (0.945, 0.403, 0.010) were used in both ethnic minority and non-minority regions (for comparison). In ethnic minority regions, hospitals are more sparsely distributed across larger geographical areas, the service area of hospitals and the hospital seeking radii of residents tend to be larger than in ethnic non-minority regions (where population and hospitals are densely distributed). This may also have led to an overestimation of the disparity of spatial accessibility within ethnic minority regions.

The current condition of healthcare accessibility in ethnic minority region is a combined result of the government policy and investment, geographical characteristic, traffic network, and population distribution pattern. Although government investment is more in ethnic minority region than in non-minority region, the road network and geography limited the location of hospitals, and further restrained the accessible healthcare services of people living in remote area (mostly minority population).

Based on our study, we identified the following problems in the supply of healthcare in ethnic minority region: 1) Doctors, health professionals, and beds are insufficient, and heavily clustered in a few sites; 2) the market mostly relies on government investment with only a small role for private investment; and 3) more primary and tertiary hospitals are needed in this region. To improve the situation in ethnic minority region, the government can focus on 1) promoting private sector investment in hospitals through beneficial policies, 2) supporting the upgrade of secondary hospitals to tertiary hospitals through favorable policies and financial support, 3) making it compulsory for qualified doctors to spend a period of time working there, and 4) push forward road construction in rural area of ethnic minority region.

Due to data limitation, our research only revealed the disparity of spatial access to healthcare services in Sichuan Province, which may not be representative of the whole condition in China. This study has focused on the comparison of spatial access to hospital services in ethnic minority and non-minority regions, without considering the inner-connection of ethnic regions, traffic networks, geography, population distribution, health seeking behavior, etc., thus resulted in some debatable parameter settings, for instance the upper limit of the shortest path analysis (120 min), the subzones of the E2SFCA method (0–10, 10–30, 30–60 min), the weights of the subzones (0.945, 0.403, 0.010), and the usage of same subzones in both ethnic minority and non-minority region. The assumption of evenly distributed population within a county is unrealistic. Populations tend to be clustered in cities or close to main roads. Thus results shown in this paper are likely to have underestimated the actual proportion of the population with access to hospitals within 2 h, and overestimated the disparity of spatial accessibility within ethnic minority and non-minority regions. We have realized the insufficiencies of our research and all these have been taken into account in our future work.

## Conclusions

In this paper, we calculated the shortest travel time and spatial accessibility of healthcare services for every 2 km*2 km resident unit in Sichuan Province based on ArcGIS network analysis tools and the E2SFCA method. Compared with the traditional population-to-provider ratio, our results better reflect actual geographical patterns. The identification of underserved regions can guide the location of new hospitals and investment in different regions. A better understanding of healthcare inequality in ethnic minority and non-minority region is useful for government officers in future planning efforts. We hope that the E2SFCA method can be further used in estimating public service accessibility (sanitation, education etc.) in China.

## Abbreviations

2SFCA: Two-Step Floating Catchment Area
CCP: Chinese Communist Party
CV: Coefficient of Variation
E2SFCA: Enhanced Two-Step Floating Catchment Area
GIS: Geographical Information System
PPR: Population-To-Provider Ratio
PRC: People’s Republic of China

## References

[1] Zang X (2008). Market reforms and Han-Muslim variation in employment in the Chinese state sector in a Chinese city. World Dev.

[2] Gustafsson B, Sai D (2014). Why is there no income gap between the Hui Muslim minority and the Han majority in rural Ningxia, China?. China Q.

[3] Liu J, Li W (2015). A nighttime light imagery estimation of ethnic disparity in economic well-being in mainland China and Taiwan (2001–2013). Eurasian Geogr Econ.

[4] Maurer-Fazio M, Hughes JW, Zhang D (2010). A Comparison of reform-era labor force participation rates of China’s ethnic minorities and Han majority. Int J Manpow.

[5] Gao W, Smyth R (2011). Economic returns to speaking ‘standard Mandarin’ among migrants in China’s urban labour market. Econ Educ Rev.

[6] Cao H (2010). Urban–rural income disparity and urbanization: what is the role of spatial distribution of ethnic groups? A case study of Xinjiang Uyghur Autonomous Region in western China. Reg Stud.

[7] Wu J (2014). The rise of ethnicity under China’s market reforms. Int J Urban Reg Res.

[8] Sautman B (2010). Scaling back minority rights?: The debate about China’s ethnic policies. Stanford J Int Law.

[9] Sautman B (1998). Affirmative action, ethnic minorities and China’s universities. Pac Rim Law Policy J.

[10] Yang Z, Wang K, Li TL (1998). Childhood diabetes in China- Enormous variation by place and ethnic group. Diabetes Care.

[11] Pan J, Liu HR, Wang XL (2015). Assessing the spatial accessibility of hospital care in Sichuan Province. China Geospatial Health.

[12] Neutens T (2015). Accessibility, equity and health care: review and research directions for transport geographers. J Transp Geogr.

[13] Fransen K, Neutens T, Maeyer PD, Deruyter G (2015). A commuter-based two-step floating catchment area method for measuring spatial accessibility of daycare centers. Health Place.

[14] Luo W, Wang F (2003). Measures of spatial accessibility to health care in a GIS environment: synthesis and a case study in the Chicago region. Environ Plan B-Plan Des.

[15] Luo W, Qi Y (2009). An enhanced two-step floating catchment area (E2SFCA) method for measuring spatial accessibility to primary care physicians. Health Place.

[16] Wan N, Zhan FB, Zou B (2012). A relative spatial access assessment approach for analyzing potential spatial access to colorectal cancer services in Texas. Appl Geogr.

[17] McGrail MR, Humphreys JS (2009). The index of rural access: an innovative integrated approach for measuring primary care access. BMC Health Serv Res.

[18] McGrail MR, Humphreys JS (2009). Measuring spatial accessibility to primary care in rural areas: Improving the effectiveness of the two-step floating catchment area method. Appl Geogr.

[19] Donaldson JA (2007). Tourism, development and poverty reduction in Guizhou and Yunnan. China Q.

[20] Jiang XJ, Liu ZL, She Q (2014). Blood pressure control rate and associated risk factors in hospitals of different grades in Chongqing. China Int J Cardiol.

[21] Yan B, Zhu D, Cheng J (2010). The status of glycemic control: a cross-sectional study of outpatients with type 2 diabetes mellitus across primary, secondary, and tertiary hospitals in the Jiangsu province of China. Clin Ther.

[22] Guo WZ, Li WZ (2009). Studying on the status of financial compensation for state-owned hospitals in Sichuan Province and its counter measures. Chin Health Serv Manage.

